# An improved protocol to study the plant cell wall proteome

**DOI:** 10.3389/fpls.2015.00237

**Published:** 2015-04-10

**Authors:** Bruno Printz, Raphaël Dos Santos Morais, Stefanie Wienkoop, Kjell Sergeant, Stanley Lutts, Jean-Francois Hausman, Jenny Renaut

**Affiliations:** ^1^Environmental Research and Innovation Department, Luxembourg Institute of Science and TechnologyBelvaux, Luxembourg; ^2^Groupe de Recherche en Physiologie Végétale, Earth and Life Institute Agronomy, Universiteì catholique de LouvainLouvain-la-Neuve, Belgium; ^3^Department for Molecular Systems Biology, University of ViennaVienna, Austria

**Keywords:** proteomics, cell wall, plant, glycosylation, EGTA

## Abstract

Cell wall proteins were extracted from alfalfa stems according to a three-steps extraction procedure using sequentially CaCl_2_, EGTA, and LiCl-complemented buffers. The efficiency of this protocol for extracting cell wall proteins was compared with the two previously published methods optimized for alfalfa stem cell wall protein analysis. Following LC-MS/MS analysis the three-steps extraction procedure resulted in the identification of the highest number of cell wall proteins (242 NCBInr identifiers) and gave the lowest percentage of non-cell wall proteins (about 30%). However, the three protocols are rather complementary than substitutive since 43% of the identified proteins were specific to one protocol. This three-step protocol was therefore selected for a more detailed proteomic characterization using 2D-gel electrophoresis. With this technique, 75% of the identified proteins were shown to be fraction-specific and 72.7% were predicted as belonging to the cell wall compartment. Although, being less sensitive than LC-MS/MS approaches in detecting and identifying low-abundant proteins, gel-based approaches are valuable tools for the differentiation and relative quantification of protein isoforms and/or modified proteins. In particular isoforms, having variations in their amino-acid sequence and/or carrying different N-linked glycan chains were detected and characterized. This study highlights how the extracting protocols as well as the analytical techniques devoted to the study of the plant cell wall proteome are complementary and how they may be combined to elucidate the dynamism of the plant cell wall proteome in biological studies. Data are available via ProteomeXchange with identifier PXD001927.

## Introduction

Cell walls are biological composites developing outside the cells and forming a rigid frame protecting the cell. Plant cell walls fulfill a wide variety of roles which differ between cell types, plants, and species (Cosgrove, 2005; Guerriero et al., 2014a,b). Cell walls are generally composed of cellulose, lignin, and hemicellulose embedded in an aqueous glue of pectins. The growth of the cell wall is further determined by the presence of minerals (in particular calcium) and the activity of enzymes and structural proteins that account for up to 10% of the mass of the wall of growing cells (Wolf et al., 2012).

Since the first study directed at the proteome of the plant cell wall, more than 55 papers (http://www.polebio.lrsv.ups-tlse.fr/WallProtDB/index.php/links) have been published and extensive research has been carried out on the model species *Arabidopsis thaliana* (Albenne et al., 2013). Nonetheless, the remarkable diversity in composition and function of the wall across cells, organs and species and the regain of interest in plant by-products in the industrial field [as source of bioethanol (Sreenath et al., 2001), building components (Nozahic et al., 2012), and biopolymers (Hühns and Broer, 2010)] foster the analysis of the cell wall proteome.

The extracellular nature of the wall and the range of binding-affinities that proteins have for the extracellular matrix make the purification of cell wall proteins in a one-step procedure difficult. The major steps made to improve the enrichment of cell wall proteins (CWPs) have previously been reviewed (Feiz et al., 2006; Jamet et al., 2008; Albenne et al., 2013). Although, CWPs enrichment can be done using non-destructive techniques which preserve membrane integrity, the use of destructive methods that require the grinding of the plant material and consequently the disruption of the plasma membranes is commonly preferred. In these protocols, CWPs are extracted from the ground plant material by washes in buffers of various ionic strengths. In 2004, Watson et al. used a washing procedure using sodium acetate, sodium chloride, and ascorbic acid followed by successive vacuum filtrations on nylon mesh membranes with sodium chloride, water, acetone, and sodium acetate (Watson et al., 2004). In 2006, Feiz et al. introduced a procedure which combines extractions with low ionic strength acidic buffers with different washes in increased sucrose concentration shown to considerably limit the contamination of the wall fraction with intracellular proteins, probably by helping the elimination of organelles and other vesicles less dense than cell wall polysaccharides (Feiz et al., 2006).

In the first studies on CWPs (Bozarth et al., 1987), proteins were extracted with a CaCl_2_ solution, later studies proposed the enrichment of CWPs and the reduction of the complexity of the extracts by using CaCl_2_, cyclohexylenedinitrilotetraacetate (CDTA), DTT, NaCl and borate buffers to sequentially extract proteins with various wall-binding affinities (Robertson et al., 1997). Quicker methods involving only a two-steps fractionation using sodium acetate buffers with CaCl_2_ or LiCl, known to be efficient extractants of CWPs, were then developed on crushed plant material (Watson et al., 2004; Feiz et al., 2006). Recently, CaCl_2_ was replaced by the chelating agent EGTA to remove the proteins associated with the pectin fraction (Verdonk et al., 2012). Regarding the high degree of variability of the cell wall across species, organs and growing conditions, a broad comparison of these protocols starting from the same initial material appears essential.

Independent of the extraction procedure, CWPs can be identified using different methods involving either 2D LC-MS/MS analysis of the total digested proteins or a separation on gels, followed by a digestion step and MS/MS analysis (Jamet et al., 2008). Although, the basic glycoproteins that are found in the cell wall may be poorly resolved on 2D-gels, this method allows the separation and the relative quantification of different isoforms of a protein and eases the identification of post-translational modifications that may have occurred during the maturation of these proteins.

In this study, the protocols used in the two major studies dealing with the alfalfa stem cell wall proteome (Watson et al., 2004; Verdonk et al., 2012) were tested and compared with a third, hybrid protocol. In this latter protocol, adapted from Verdonk et al. (2012) and Feiz et al. (2006), a sequential three-step extraction based on low ionic strength buffers with additional CaCl_2_, EGTA, and LiCl is performed. The three protocols are compared by a direct analysis of the digested proteins using LC-MS/MS. Two-Dimensional electrophoresis were further carried out on the extracts from the hybrid protocol to highlight how this technique can complement LC-MS/MS analysis of plant CWPs.

## Methods

### Plant material

Alfalfa stems (*Medicago sativa* L.) were harvested from a local field (49°33′39.1″N, 5°41′38.0″E, Musson, Belgium) in early spring 2014. After removal of the leaves, stems were ground to a homogeneous powder in a mortar filled with liquid nitrogen. In this study, about 30 g of field-grown fresh alfalfa stems were frozen in liquid nitrogen, ground, and divided in 3 × 2 (protocols × replicates) samples of about 5.0 g and stored at −80°C prior to analysis.

### Cell wall protein extraction

**Figure 1:**
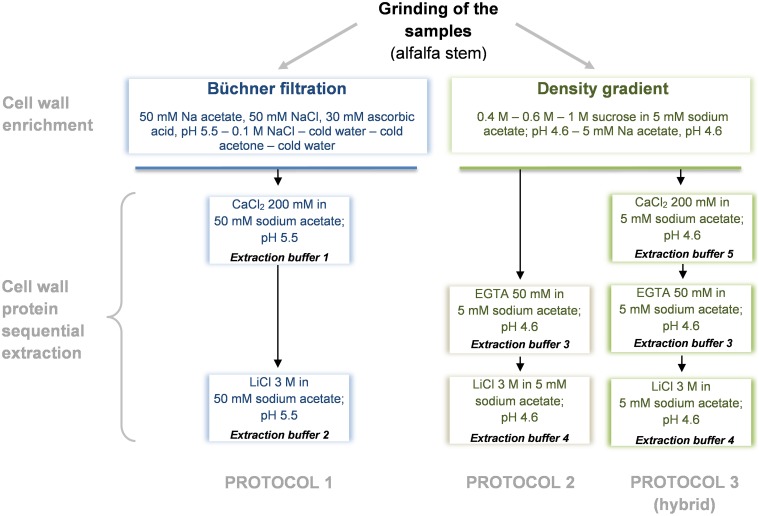
**Diagram overview of the cell wall protein extraction protocols**. Protocol 1, adapted from Watson et al. (2004); Protocol 2, adapted from Verdonk et al. (2012); Protocol 3 (hybrid), adapted from Verdonk et al. (2012) and Feiz et al. (2006).

CWPs were extracted as described in Watson et al. (2004) (protocol 1) and in (Verdonk et al., 2012) (protocol 2). A third hybrid protocol adapted from Feiz et al. (2006) and Verdonk et al. (2012) was established (protocol 3) to analyze the role of the chelating agent EGTA used by Verdonk et al. (2012). Each extraction procedure was carried out in 2 replicates and performed as summarized in Figure 1. Minor modifications were done to the previously described protocols, these were done to focus on the differences induced by the extraction and less on differences in the first steps of the sample preparation. Another adaptation done was the use of the same, 2D-DIGE compatible, buffer for the final resolubilization of the extracted proteins.

### Protocol 1 (adapted from Watson et al., 2004)

The CWPs isolation and extraction were adapted from Watson et al. (2004), Modifications include the absence of PVPP in the grinding buffer (Watson and Sumner, 2007), the filtering of the plant material through 30 μm2 pore size nylon mesh and the resuspension of the proteins in 7 M urea, 2 M thiourea, 2% w/v CHAPS, 30 mM Tris. The plant material was placed in a 50 ml Falcon® tube with 10 ml of buffer (50 mM Na acetate, 50 mM NaCl, and 30 mM ascorbic acid, pH 5.5, 4°C). After vigorous shaking (24 Hz, 2 min), the slurry was filtered through a nylon mesh membrane (30 μm^2^ pore size) under vacuum and washed sequentially with (a) 100 mL of 50 mM Na acetate, 50 mM NaCl, and 30 mM ascorbic acid, pH 5.5, 4°C, (b) 50 mL of NaCl (0.1 M, 4°C), (c) 100 mL of cold water (4°C), (d) 250 mL of cold acetone (4°C), and (e) 100 mL of cold water. The retentates were transferred to 30 ml tubes prior to protein extraction.

Cell wall protein extraction was carried out by resuspending the retentates in 7.5 mL of extraction buffer 1 (50 mM Na acetate, 200 mM CaCl_2_, pH 5.5, 4°C). Samples were placed on a rocking platform (30 min, 4°C), centrifuged (10,000 g, 15 min, 4°C) and the supernatants were saved. The pellets were re-extracted once with extraction buffer 1 and both supernatants of the same sample were pooled to form the CaCl_2_ fraction.

The pellets were resuspended in 15 mL of extraction buffer 2 (50 mM Na acetate, 3 M LiCl, pH 5.5, 4°C), placed on a rocking platform (overnight, 4°C) and centrifuged (10,000 g, 15 min, 4°C). The supernatants were saved, forming the LiCl fraction of the samples.

### Protocol 2 (adapted from Verdonk et al., 2012)

Cell wall proteins were extracted as presented in Verdonk et al. (2012) with the following adaptations. Only 5 g of fresh matter were used for extraction, proteins were precipitated and washed using ReadyPrep^™^ 2-D Cleanup Kit (Bio-Rad) and proteins were resuspended in 7 M urea, 2 M thiourea, 2% w/v CHAPS, 30 mM Tris.

Briefly, the plant material was placed in a 50 ml Falcon® tube with 20 mL of buffer A (5 mM Na acetate, 0.4 M sucrose, pH 4.6, 4°C), shaken vigorously (24 Hz, 2 min) and placed on a rocking platform (overnight, 4°C). Samples were then centrifuged (1000 g, 15 min, 4°C) and supernatants were discarded. Both pellets were resuspended in 10 mL of buffer B (5 mM Na acetate, 0.6 M sucrose, pH 4.6, 4°C) and placed on a rocking platform (30 min, 4°C) and centrifuged again (1000 g, 15 min, 4°C). Supernatants were discarded. This washing step was repeated respectively with buffer C (5 mM Na acetate, 1 M sucrose, pH 4.6, 4°C) and twice with buffer D (5 mM Na acetate, pH 4.6, 4°C). The isolated cell wall fractions (pellet) were then transferred to 30 mL tubes.

Proteins were extracted with 10 mL of extraction buffer 3 (5 mM Na acetate, 50 mM EGTA, pH 4.6) and samples were shaken vigorously at 37°C for 1 h. After centrifugation (10,000 g, 15 min, 4°C), supernatants were saved. This extraction step was repeated twice and all supernatants were pooled, leading to the EGTA fraction.

The remaining pellet was resuspended in 10 mL of extraction buffer 4 (5 mM Na acetate, 3 M LiCl, pH 4.6, 4°C), placed on a rocking platform (overnight, 4°C) and centrifuged (10,000 g, 15 min, 4°C). Supernatants were saved and pellets were re-extracted twice using the same procedure with a shaking step lasting at least 8 h. All supernatants were pooled, leading to the LiCl fraction.

### Protocol 3 (adapted from Feiz et al., 2006 and Verdonk et al., 2012)

The isolation of the cell wall fraction was carried out using sequential washes in increased sucrose concentration as described in protocol 2.

The proteins from the isolated cell wall fraction (pellet) were extracted with 7.5 mL of extraction buffer 5 (5 mM Na acetate, 200 mM CaCl_2_, pH 4.6, 4°C) and placed on a rocking platform (30 min, 4°C). Samples were then centrifuged (10,000 g, 15 min, 4°C) and supernatants saved. This step was repeated once and supernatants were pooled, leading to the CaCl_2_ fraction.

Proteins were further extracted with 10 mL of extraction buffer 3 (5 mM Na acetate, 50 mM EGTA, pH 4.6) and shaken vigorously at 37°C for 1 h. After centrifugation (10,000 g, 15 min, 4°C), supernatants were saved. This extraction step was repeated twice and supernatants were pooled leading to the EGTA fraction.

The remaining pellet was finally resuspended in 15 mL of extraction buffer 4 (5 mM Na acetate, 3 M LiCl, pH 4.6, 4°C), placed on a rocking platform (overnight, 4°C) and centrifuged (10,000 g, 15 min, 4°C). Supernatants were saved, forming the LiCl fraction.

### Concentration and desalting of the extracts

Each cell wall enriched protein fraction was concentrated by using an Amicon Ultra-15 10K Centrifugal Filter Device (Millipore) and centrifuged (4700 g, 4°C) until reaching a final volume of approximately 200 μL. Proteins were further washed and desalted with a ReadyPrep 2-D Cleanup Kit (Bio-Rad) according to manufacturer instructions. After drying, proteins were solubilized in 100 μL labeling buffer (7 M urea, 2 M thiourea, 2% w/v CHAPS, 30 mM Tris) and protein concentrations were assessed by using the Bradford protein assay with BSA as standard (Bradford, 1976).

### Protein analysis

### SDS-page

The reproducibility of the extractions was assessed by SDS-PAGE. Proteins, 20 μg of each sample (2 replicates by fraction) were loaded on Criterion^™^ XT precast 1D gel 12% Bis-Tris, 12 + 2 wells, 45 μL, 1.0 mm (Bio-Rad) according to manufacturer's instructions. Proteins were allowed to migrate for 1 h at a constant voltage of 200 V. After migration, gels were stained for 45 min with 100 ml of InstantBlue solution (Expedeon). Gels were rinsed twice with deionized water and scanned using a Typhoon FLA 9500 scanner (GE Healthcare).

### Focus on the hybrid three-steps protocol using 2-dimensional gel electrophoresis

#### Protein separation

CWPs, 50 μg *per* fraction (1 replicate), were mixed with 9 μL Servalyte, pH 3-10 (Serva Electrophoresis GmbH) and 2.7 μL of Destreak Reagent (GE Healthcare) and volumes were completed to 450 μL with lysis buffer (7 M urea, 2 M thiourea, 0.5% (w/v) CHAPS). Samples were loaded onto Immobiline^™^ DryStrip 3-10 NL, 24 cm (GE Healthcare) during overnight rehydration.

Isoelectric focusing was carried out in a five step-program: (1) constant 100 V for 3 h, (2) linear gradient from 100 to 1000 V for 4 h, (3) constant 1000 V for 6 h, (4) linear gradient from 1000 to 10,000 V for 6 h, and (5) constant 10,000 V until reaching a total of 95,000 Vh. During IEF, the current was limited to 75 μA *per* strip.

Strips were then equilibrated 15 min in equilibration buffer (Serva Electrophoresis GmbH) complemented with 6 M Urea and 1% w/v DTT and further 15 min in equilibration buffer complemented with 6 M Urea and 2.5% w/v IAA. Strips were loaded on 2D-HPE^™^ Large-Gels NF 12.5% (Serva Electrophoresis GmbH) and electrophoresis was carried out using an HPE^™^ Tower System according to manufacturer's instructions. After the front reached the bottom of the gel, the gels were placed in fixation solution containing 15% v/v ethanol complemented with 1% (m/v) of citric acid. Gels were subsequently placed for 90 min in a LavaPurple (Serva Electrophoresis GmbH) staining solution (0.005% v/v) containing 100 mM NaOH, 100 mM boric acid. After staining, gels were washed twice with 15% EtOH for 15 min, acidified again for 15 min in fixation solution and rehydrated in deionized water. Gels were subsequently scanned at 473 nm using a Typhoon FLA 9500 scanner (GE-Healthcare), and quantitative analysis was carried out using the DeCyder software (v7.0, GE-Healthcare).

#### Protein digestion and analysis

Following spot detection, all visible spots of each of the 3 gels were stored in a picklist and picked with an Ettan Spotted Picker (GE Healthcare). Digestion and MALDI spotting were carried out using a Freedom EVO II workstation (Tecan). Briefly, gels plugs were washed for 20 min in a 50 mM ammonium bicarbonate solution in 50% v/v MeOH/MQ water and dehydrated for 20 min with 75% ACN. After dehydration, proteins were digested with trypsin Gold (Promega), 8 μl of a solution containing 5 ng/μL trypsin in 20 mM ammonium bicarbonate (overnight, 37°C). After digestion, peptides were extracted from the gel plugs with 50% v/v ACN containing 0.1% v/v TFA and dried. Peptides were then solubilized in 2 μL of 50% v/v ACN containing 0.1% v/v TFA and 0.7 μL was spotted on MALDI-TOF targets. A volume of 0.7 μL of 7 mg/mL α-cyano-4-hydroxycinnamic acid in 50% v/v ACN containing 0.1% v/v TFA was added. A MALDI peptide mass spectrum was acquired using the AB Sciex 5800 TOF/TOF (AB Sciex), and the 10 most abundant peaks, excluding known contaminants, were automatically selected and fragmented. MS analyses were carried out as described by Printz et al. (2013). MS and MS/MS spectra were submitted for NCBInr database-dependent identification using the taxonomy *viridiplantae* (http://www.ncbi.nlm.nih.gov) downloaded on September 23, 2013 and containing 32,770,904 sequences on an in-house MASCOT server (Matrix Science, www.matrixscience.com). A second search was carried out against an EST *fabacea* database downloaded on December 17, 2013 and containing 19,932,450 sequences. The parameters used for these searches were mass tolerance MS 100 ppm, mass tolerance MS/MS 0.75 Da, fixed modifications cysteine carbamidomethylation, and variable modifications methionine oxidation, double oxidation of tryptophan, and tryptophan to kynurenine. Proteins were considered as identified when at least two peptides passed the MASCOT-calculated 0.05 threshold scores (respectively a score of 50 for all NCBI *viridiplantae* queries and 57 for the EST *fabacea* queries).

### Liquid chromatography

#### Protein digestion

The digestion of proteins was performed using Amicon Ultra-4 10K Centrifugal Filter Devices (Millipore) (Abdallah et al., 2012). CWPs, 25 μg *per* fraction (1 replicate), were reduced for 20 min in 200 μL 10 mM DTT dissolved in 100 mM ammonium bicarbonate. After centrifugation, (30 min, 4700 g, 4°C) the sample was washed with 200 μL of 100 mM ammonium bicarbonate and again centrifuged. The reduced proteins (at the top of the filter) were alkylated with 50 mM iodoacetamide in 100 μL of 100 mM ammonium bicarbonate for 30 min in the dark and after centrifugation washed twice with 100 μL 100 mM ammonium bicarbonate. After the last centrifugation, 50 μL of trypsin Gold (Promega), 5 ng/mL trypsin in 50 mM ammonium bicarbonate, was added and the filter device incubated overnight at 37°C. Following digestion, 100 μL of deionized H_2_O were added, filter devices were centrifuged (40 min, 4700 g, 4°C), and the peptides collected at the bottom of the tube. The mixture of peptides was dried under vacuum and solubilized in 45 μL of a solution containing 2% v/v ACN and 0.1% v/v formic acid.

#### Peptide separation and analysis

A volume of 5 μL of the extracted peptides were desalted and separated by reverse phase separation using an Eksigent nano 1DLC (AB Sciex) coupled with a LTQ-OrbiTrap XL mass spectrometer (Thermo scientific) operated with Xcalibur (2.0.7 SP1). Peptide desalting was carried out on C18 OMIX tips (100 μl, Agilent Technologies) and separation was carried out at a flow rate of 400 nl.min^−1^ on a Peptide ES-C18 column (15 × 0.1 mm, 2.7 μm; Sigma-Aldrich) using a linear binary gradient (solvent A: 0.1% formic acid (FA); solvent B: 80% ACN 0.1% FA). MS and MS/MS analyses were performed online, in data-dependent mode with automatic switching between MS and MS/MS. Full scan MS spectra (300–1500 m/z) were acquired at 30,000 (m/z 400) resolution. Internal mass calibration was performed using Cyclomethicone (m/z 371.101230) as lock mass. Dynamic exclusion was enabled with exclusion size list of 500 and exclusion duration of 90 s. The eight most intense precursors were selected for subsequent fragmentation with normalized collision energy of 35%. Fragmentation spectra were acquired in the ion trap with an isolation window of 2.0 m/z, a target value of 1000, an activation Q of 0.25 and an activation time of 30 ms.

CID spectra were processed in an in-house Mascot server (Version 2.1, Matrix Science, www.matrixscience.com, London, UK) using Proteome Discoverer (version 1.4.0.288, Thermo scientific) by searching against the NCBInr database using the taxonomy *viridiplantae* (http://www.ncbi.nlm.nih.gov) downloaded on June 06, 2014 and containing 40,910,947 sequences. The searches were performed with the following parameters: used enzyme: trypsin, 2 missed cleavages, mass accuracy precursor: 10 ppm, mass accuracy fragments: 0.8 Da, fixed modifications: Carbamidomethyl (C), dynamic modifications: Dioxidation (W), Gln->pyro-Glu (N-term Q), Glu->pyro-Glu (N-term E), Oxidation (HW), Trp-> Kynurenin (W). Identifications were filtered using the following settings; high peptide confidence (minimum confidence: 95%, peptide decoy database search: Target FDR (Strict): 0.01; Target FDR (Relaxed): 0.05, Validation based on: *q*-Value), with minimum two peptides per protein. The mass spectrometry proteomics data have been deposited to the ProteomeXchange Consortium (Vizcaino et al., 2014) via the PRIDE partner repository with the dataset identifier PXD001927 and 10.6019/PXD001927. Finally, all identifications obtained based on the NCBI database were matched on the *Medicago truncatula* reference genome using an online platform available at http://plantgrn.noble.org/LegumeIP/v2/blasttranscript.jsp. Sequences were imported as FASTA sequences and blasted to the Mt4.0v1_GenesCDSSeq_20130731_1800 database. Searches were performed with an *E*-value cut-off of 1e-04 and blast results were accepted in case more than 50% identity was reported.

### Protein localization

Proteins were considered to be secreted in case the 2 servers SignalP 4.1 (http://www.cbs.dtu.dk/services/SignalP) and TargetP 1.1 (http://www.cbs.dtu.dk/services/TargetP) predicted the presence of a signal peptide cleavage site and an extracellular location with the standard search parameters.

## Results and discussion

In 2006, Feiz et al. performed a comparative analysis of CWPs isolation protocols (Feiz et al., 2006). This comparison, based on published data, remains theoretical since all protocols were not tested in the same conditions, with a same analytical method and on a same initial plant material. So far two different protocols have been applied to study the cell wall proteome of alfalfa stems, both start with a purification of the cell wall followed by two steps of cell wall protein extraction (Watson et al., 2004; Verdonk et al., 2012). Watson et al. (2004) proposed the sequential use of 200 mM CaCl_2_ and 3 M LiCl in 50 mM sodium acetate buffers and succeeded to limit the contamination with intracellular proteins to less than 50%. In the second strategy, described by Verdonk et al. (2012), CWPs are sequentially extracted with 50 mM EGTA and 3 M LiCl in 5 mM sodium acetate buffers and the percentage of proteins predicted to be targeted to the cell wall increased significantly to reach about 70%. Although, the sequential use of CaCl_2_ and LiCl buffers to extract CWPs and glycosylated proteins has been frequently reported (Irshad et al., 2008; Day et al., 2013; Calderan-Rodrigues et al., 2014), the use of a chelating agent as first extractor as proposed by Verdonk et al. is rarely depicted.

In our study, we compare these two previously published methods with a third “hybrid” protocol based on the sequential use of low ionic strength buffers (5 mM sodium acetate) complemented with 200 mM CaCl_2_, 50 mM EGTA, and 3 M LiCl respectively. This three-steps fractionation should first allow the release of the most loosely attached proteins by saturating the pectin-fraction with Ca^2+^ ions. The subsequent use of the chelating agent EGTA, that exhibits a high affinity for Ca^2+^ ions, loosens the pectin network and frees up proteins associated with it. Finally, the last extraction with a high concentration of LiCl should release proteins that are more tightly bound to the wall matrix (Verdonk et al., 2012). The isolation of the cell wall and washing were performed according to Watson et al. (2004) for protocol 1—or using the washes in different sucrose concentrations proposed by Feiz et al. (2006) for the protocols 2 and 3. For each protocol and fraction, the total mass of proteins extracted *per* g of fresh weight is presented in Table 1.

**Table 1:** **Amount of proteins extracted by fraction and by protocol**.

	**Protocol 1**	Mass of extracted proteins (in μg/g FW)^*^	
Adapted from	Watson et al., 2004	Sample 1	Sample 2
Extraction buffers	50 mM Na acetate, 200 mM CaCl_2_, pH 5.5	41.6	47.4
	50 mM Na acetate, 3 M LiCl, pH 5.5	60.0	36.8
	Mass of proteins isolated per g FW	101.6	84.2
	**Protocol 2**	Mass of extracted proteins (in μg/g FW)^*^	
Adapted from	Verdonk et al., 2012	Sample 3	Sample 4
Extraction buffers	5 mM Na acetate, 50 mM EGTA, pH 4.6	25.5	27.6
	5 mM Na acetate, 3 M LiCl, pH 4.6	126.0	116.8
	Mass of proteins isolated per g FW	151.5	144.4
	**Protocol 3 - hybrid**	Mass of extracted proteins (in μg/g FW)^*^	
Adapted from	Feiz et al., 2006 and Verdonk et al., 2012	Sample 5	Sample 6
Extraction buffers	5 mM Na acetate, 200 mM CaCl_2_, pH 4.6	156.0	171.5
	5 mM Na acetate, 50 mM EGTA, pH 4.6	26.0	28.9
	5 mM Na acetate, 3 M LiCl, pH 4.6	50.0	50.9
	Mass of proteins isolated per g FW	232.0	251.3

*Proteins were extracted from approximately 5 g of fresh alfalfa stem crushed into powder in liquid nitrogen*.

* *The mass is expressed in μg per g of fresh material*.

**Figure 2:**
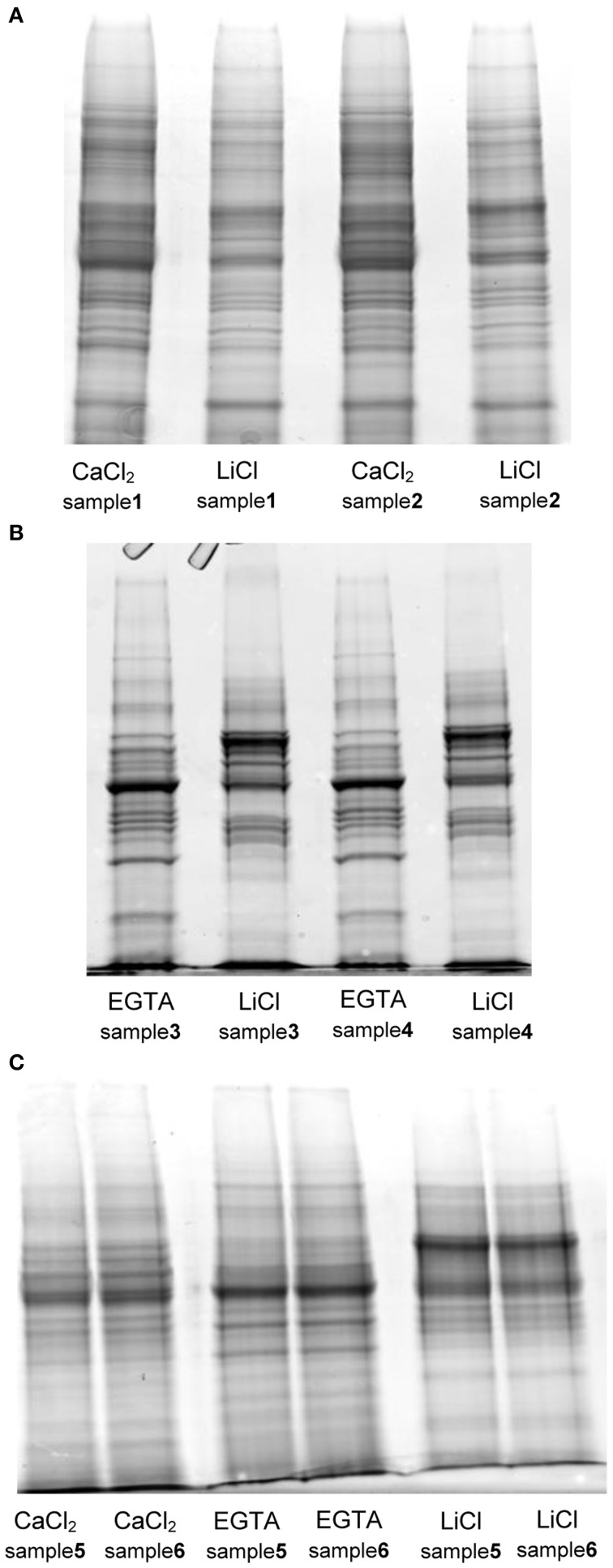
**SDS-PAGE protein profiles of the different fractions extracted with the tested protocols. (A)** protocol 1, adapted from Watson et al. (2004) **(B)** protocol 2, adapted from Verdonk et al. (2012) **(C)** protocol 3 (hybrid), adapted from Verdonk et al. (2012) and Feiz et al. (2006): each lane was loaded with 20 μg of proteins on Criterion^™^ XT precast 1D gel 12% Bis-Tris (Bio-Rad). The gels were stained with Coomassie blue (InstantBlue, Expedeon).

As already described by Feiz et al. (2006), the use of NaCl salt in an early step of the protocol, as proposed by Watson et al. (2004), decreased the amount of proteins extracted and thus potentially the number of CWPs present in the extract. The difference in the amount of protein extracted in the two replicates of protocol 1 is remarkable, and in our opinion stems from the fact that the isolation of the cell wall fraction using a Büchner filter is more difficult to control compared to the use of sucrose washes. In comparison the washes with sucrose led to extract the highest amount of proteins. The mass of extracted proteins was however significantly higher for the three-step hybrid protocol. For each protocol, the SDS-PAGE profiles of the two replicates were compared (Figure 2). Both fractions (CaCl_2_ and LiCl) of the protocol adapted from Watson et al. (2004) have a similar protein profile although the intensity of some gel bands varies between fractions (Figure 2A). In contrast, the use of EGTA followed by LiCl in protocol 2 (adapted from Verdonk et al., 2012) and 3 (adapted from Feiz et al., 2006 and Verdonk et al., 2012) results in distinct protein patterns between the fractions (Figure 2B). This indicates that the use of the chelating agent allows the extraction of set of proteins different from those extracted by CaCl_2_. The similarity between the replicate profiles indicates the reproducibility of the different extraction steps.

### LC-MS/MS shotgun analysis

To screen the efficiency of the protocols in extracting alfalfa stem CWPs 1 of the 2 replicates was selected, the extracts were digested with trypsin and analyzed by LC-MS/MS. The total number of identified proteins, all fractions taken together, was the highest (458) for samples treated with protocol 2, which uses EGTA followed by LiCl (Verdonk et al., 2012). For the hybrid protocol a total of 331 proteins were identified while protocol 1 allowed the identification of 106 proteins. The highest ratio “cell wall protein”/“total proteins” was obtained with the protocol 1, i.e., 83% of the proteins identified in the two fractions were predicted to be secreted. However, since the total number of identified proteins is low, only 88 (out of 106) potential CWPs were identified using this method. In contrast, when proteins were extracted according to protocol 2, the number of proteins predicted to target the secretory pathway reached 212 (out of 458), which is in the range of what was identified in the original paper of Verdonk et al. (188 out of 272). Compared to the specificity for CWPs described in the original publication, in the current dataset the number of cytosolic contaminants was rather high; 54% of the identified proteins vs. 31% in the original study. The higher number of identified non-cell wall proteins in protocol 2 in comparison with the hybrid protocol is however not surprising. Indeed, compared to the other extraction buffers the amount of protein extracted with the EGTA-complemented buffer is relatively low (Table 1, protocol 2 first step and protocol 3 second step). In addition, the bulk of the proteins identified in the EGTA fraction of protocol 2 are non-cell wall proteins. This, together with the fact that all analyses start with a defined amount of protein (5 μl for LC-MS/MS analysis and 50 μg for gel-based analysis), makes that non-cell wall proteins are more abundant in protocol 2 to allow a significant identification using mass spectrometry.

The use of the three-steps fractionation in the hybrid method led to a relative decrease of the number of identified non-CWPs to 30%. Altogether, analysis of the identified sequences with SignalP and TargetP designated 242 out of the 331 proteins identified in the fractions of the hybrid protocol (73.1%) as putative cell wall proteins; indicating that the latter protocol combines a good selectivity for cell-wall proteins with a high yield of extraction. Globally, 601 different NCBInr accessions were identified in this study, among which 322 were predicted to be cell wall located (Supplementary Table 1).

**Figure 3:**
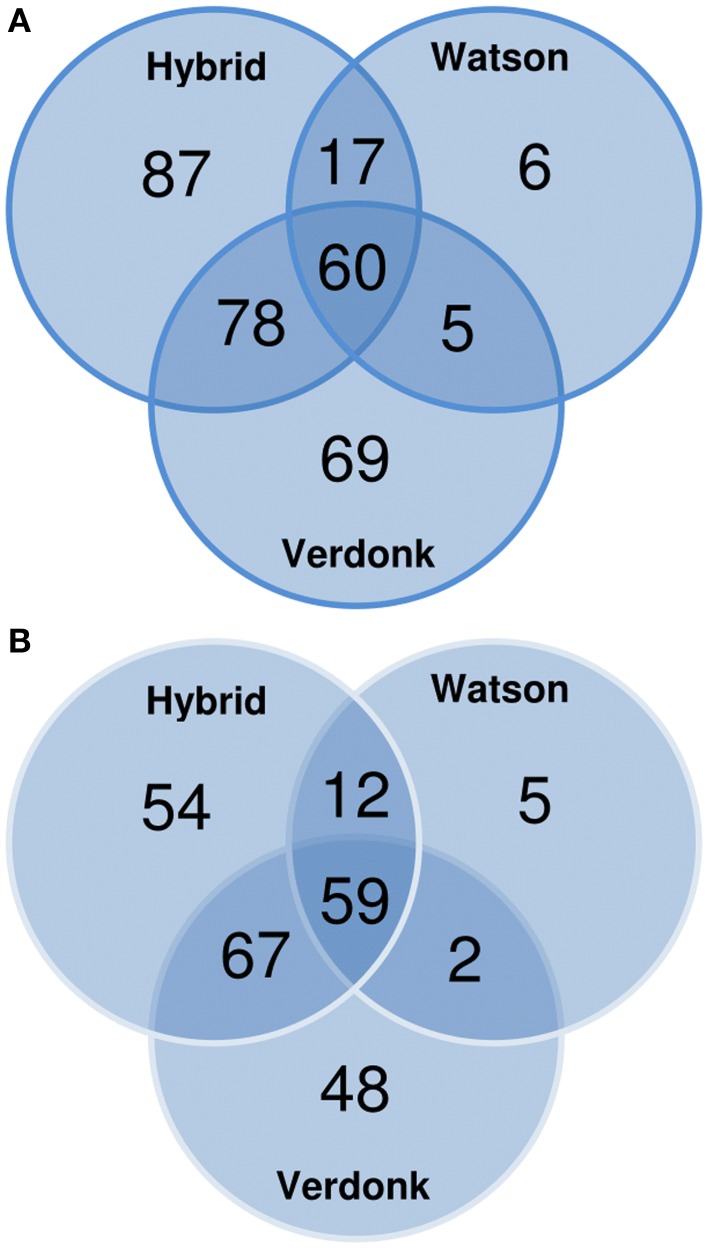
**Venn diagrams showing the repartition of the cell wall proteins (CWPs) identified by LC-MS/MS analysis according to (A) the NCBInr accessions and (B) the non-redundant Mt4.0v1 accessions obtained after blasting the NCBInr accessions on the *Medicago truncatula* genome**.

One shortcoming of the use of the NCBInr database is the redundancy, leading to an overestimation of the real number of CWPs identified. To circumvent this, all NCBInr gene identifiers were matched to the Mt4.0v1 *Medicago truncatula* reference genome. The 322 (NCBI gi) putative CWPs found in this study matched to 247 (~77%) non-redundant *M. truncatula* Gene Accessions. The effectiveness of the different protocols was then determined by analyzing the overlap between the total number of identified CWPs (the set of 247 *M. truncatula* matched gene accessions) and the number of proteins identified in each of the protocols. The three-steps fractionation scored best in this comparison, allowing the identification of 192 of the 247 *M. truncatula* Gene Accessions (77.7%). The protocol proposed by Verdonk et al. (2012) presented a similar result (176; ~71.3%), whereas the protocol described by Watson et al. (2004) allowed only the identification of 31.2% (78) of the total set of non-redundant *M. truncatula* gene accessions found in this study (Figure 3). Surprisingly, more than 43% of the *M. truncatula* gene accessions were only identified in extracts from one of the 3 protocols, suggesting that combining different protocols to study a sample increases the number of identified CWPs (Figure 3).

**Figure 4:**
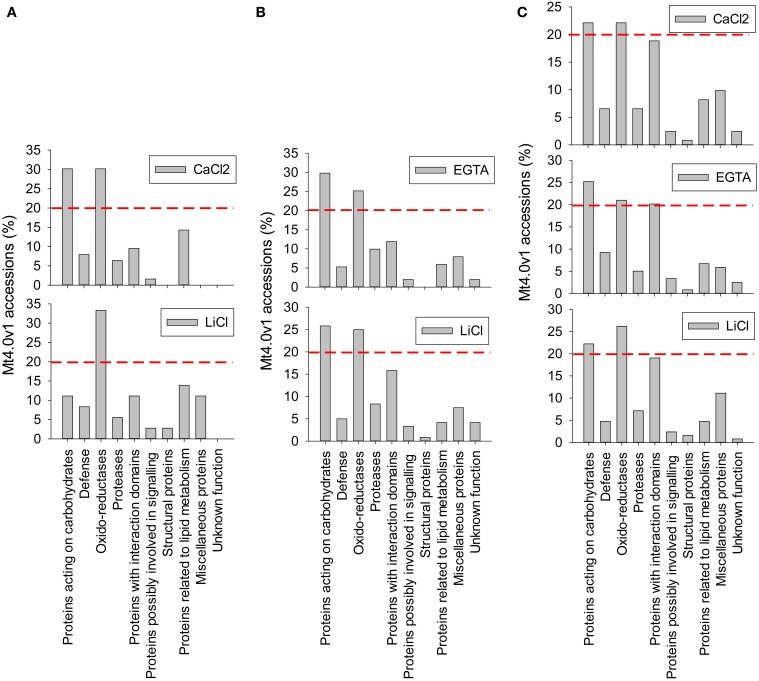
**Functional classification of the cell wall proteins (CWPs) identified by LC-MS/MS analysis of the different fractions**. Calculations were made relatively to the number of non-redundant Mt4.0v1 accessions identified in each fraction. A red line was arbitrary drown at the level 20% to allow a rapid comparison of the most represented classes of proteins in each fraction. **(A)** protocol 1, adapted from Watson et al. (2004) **(B)** protocol 2, adapted from Verdonk et al. (2012) **(C)** protocol 3 (hybrid), adapted from Verdonk et al. (2012) and Feiz et al. (2006). Note the differences observed in the CaCl_2_ fraction of the protocol adapted from Watson et al. (2004) **(A)** and the CaCl_2_ fraction of the “hybrid” protocol **(C)**. These differences are related with the 2 different procedures of cell wall isolation, the first based on filtration on Büchner devices, the second being based on washes in various sucrose concentrations.

From the LC-MS/MS analysis it is concluded that the protocols adapted from Verdonk et al. and the hybrid protocol, respectively protocols 2 and 3, have a higher efficiency in extracting alfalfa stem CWPs. Table 2 and Figure 4 show the functional classification of the cell wall proteins identified by LC-MS/MS analysis of the different extracts, the functional classification was done based on previously listed functional classes (Jamet et al., 2008). Classification was performed according to the list of domain hits proposed in the NCBI blast after amino acid sequence comparison (http://blast.ncbi.nlm.nih.gov/Blast.cgi), from protein functional analysis using the web tool (http://www.ebi.ac.uk/Tools/pfa/iprscan5) and from published data. However, the class “defense” was added, although most of the proteins from this category may also be classified as “proteins with interaction domains” or “proteins acting on carbohydrates.” Both protocols allowed the identification of more than 200 putatively-secreted proteins based on the NCBInr database search, most of them being classified in the functional classes of the oxido-reductases and in the class of proteins acting on carbohydrates (Table 2). The protocol adapted from Verdonk et al. however led to the identification of a higher proportion of non-CWPs.

**Table 2:** **Functional classification of the predicted cell wall proteins (CWPs) detected by LC-MS/MS analyses and based on the non-redundant Mt4.0v1 accessions**.

**Functional classification**	**Total (%)**	**% Range**
Proteins acting on carbohydrates	26.3	24.5 – 27.8
Oxido-reductases	21.9	20.8 – 28.2
Proteins with interaction domains	14.2	10.3 – 16.7
Proteases	8.9	7.7 – 9.7
Miscellaneous proteins	8.9	5.1 – 9.9
Defense	7.3	5.1 – 7.7
Proteins related to lipid metabolism	6.9	5.7 – 11.5
Proteins possibly involved in signaling	2.4	2.6 – 3.1
Unknown function	2	0 – 2.8
Structural proteins	1.2	0.6 – 1.3

*Percentages are calculated respectively relatively to the total number of accessions identified in the study (247, column “Total”), or relatively to the number of accession found by fraction in each protocol [192 (Hybrid), 176 (adapted from Verdonk et al.) and 78 (adapted from Watson et al.), column “% Range”]. Only the minimal and the maximal values from this last calculation are presented*.

### 2-DE analysis

Given the lowest proportion of cellular contaminants and the highest yield of CWPs the three-steps fractionation using washings with sucrose and extraction with CaCl_2_, EGTA, and LiCl complemented buffers was selected for a more detailed proteomic characterization of the 3 fractions using 2D-PAGE.

**Figure 5:**
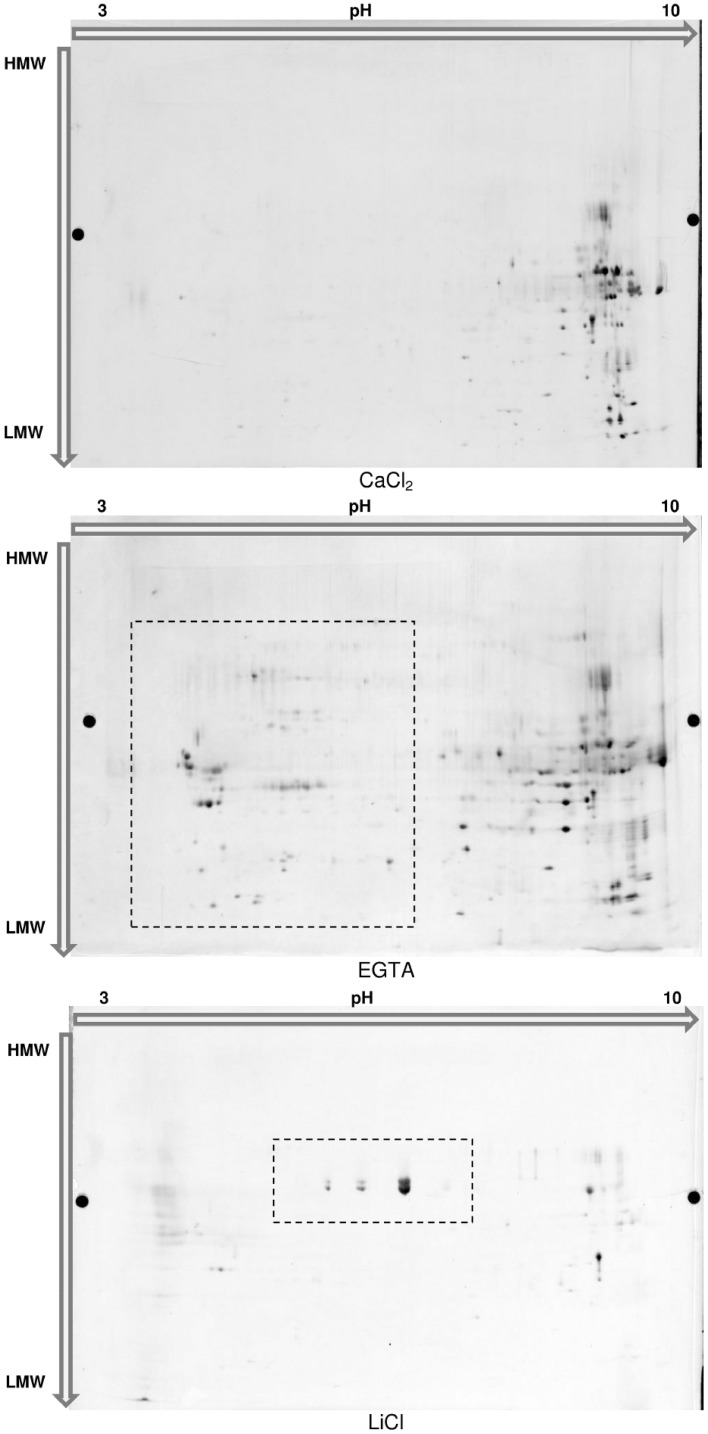
**2D-Electrophoresis of the proteins extracted in each fraction of the hybrid protocol**. Proteins were separated on Immobiline^™^ DryStrip 3-10 NL, 24 cm (GE Healthcare) and further migrated on 2D-HPE^™^ Large-Gels NF 12.5% (Serva Electrophoresis GmbH). Proteins were post-stained with LavaPurple (Serva Electrophoresis GmbH).

The main benefit of this technique would be to visualize different isoforms of a protein in case these isoforms present variations in their isoelectric point or mass. Confirming the 1D-analysis, the 2D-profile of each fraction showed clear distinctions (Figure 5). The 172 spots detected in the CaCl_2_ fraction are mainly located in the basic part of the gel. The sequential extractions using EGTA and LiCl complemented buffers allowed the detection of respectively, 207 and 59 spots. In particular, a supplemental acidic cluster of proteins is present in the EGTA fraction. The final cell wall protein fraction (LiCl) presents a limited number of spots, most of them being localized at pH ranging from about 5–8 and being not present in the 2 previous fractions.

**Figure 6:**
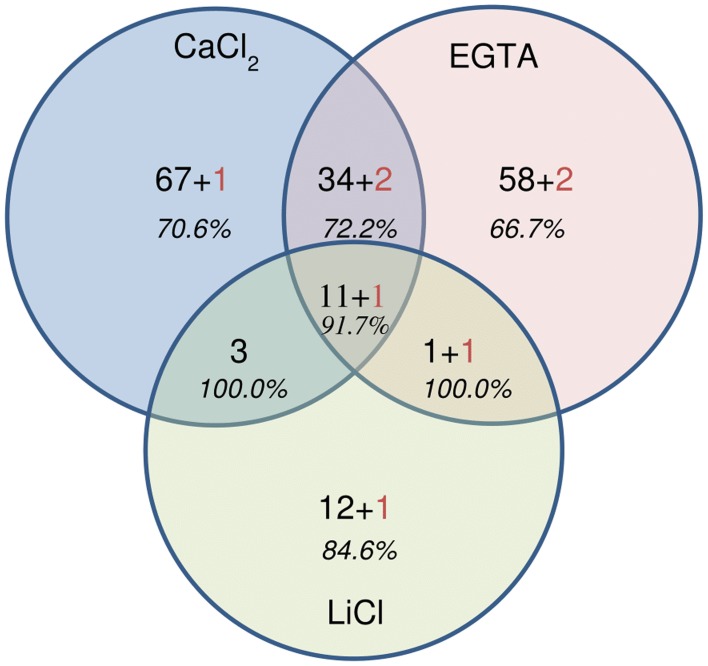
**Venn diagram showing the repartition of the 194 proteins identified by MALDI-TOF-TOF**. Numbers in black correspond to the number of plant proteins, numbers in red indicate the number of fungal proteins. In italics, the percentage of cell wall proteins (CWPs) is indicated.

For each fraction of the hybrid protocol, all spots were picked, the proteins digested and the peptides analyzed using MALDI-TOF-TOF (all the identified peptides are represented in Supplementary Tables 2a–d). A set of 194 NCBInr accessions were identified significantly, among which 186 originated from plants and 8 from fungi (Supplementary Table 2a and Figure 6). The identification of fungal proteins is not surprising since these are field-grown samples and the fungi from which proteins are identified are known pathogens of alfalfa. Interestingly, most of these fungal proteins (5 out of 8) were also predicted to carry a signal peptide which targets the protein to the secretory pathway. Although, some spots contained more than one protein, a unique and significant protein was identified in 87 out of 172 spots of the CaCl_2_ fraction, 141 out of 207 in the EGTA fraction and 43 out of 59 in the LiCl fraction.

Confirming the results obtained with LC-MS/MS, a large majority of the accessions identified by MALDI TOF-TOF [74.8% (CaCl_2_ fraction), 73.1% (EGTA fraction), and 88.9% (LiCl fraction)] was predicted to carry a signal peptide for targeting to the cell wall. As already mentioned when discussing the LC-MS/MS results, some proteins, notably pectin methylesterases (PME), may not have a classical signal peptide but only a putative transmembrane (TM) domain. In PME, the presence of a TM domain in absence of peptide signal might nonetheless be sufficient to target the protein to the cell wall (Pelloux et al., 2007). Other proteins without a predicted signal peptide identified in the current study may similarly be localized in the cell wall, suggesting that the proportion of CWPs might be underestimated (Albenne et al., 2013).

In contrast to the results obtained with LC, a higher proportion of the predicted cell wall proteins was specific to one of the fractions, for instance 57.1% of the proteins identified in the CaCl_2_ fraction were only identified in this fraction. For the EGTA and LiCl fractions this proportion is 54.5 and 43.3% respectively, while LC-MS/MS analysis gave 24.1, 22.6, and 35.6% respectively (Table 3). Such differences may be explained by the lower sensitivity of the 2DE-appraoches in detecting proteins that are of low abundance in the extract. This interpretation is confirmed by the significantly lower number of total proteins identified on the 2DE-gels (194 NCBInr identifiers), in comparison with the 331 NCBI identifiers obtained from the LC-MS/MS analysis.

**Table 3:** **Information about the cell wall proteins (CWPs) detected in the fractions of the hybrid protocol by 2D-electrophoresis**.

	**CaCl_2_**	**EGTA**	**LiCl**
Number of significantly identified predicted cell wall proteins	86	76	24
Total number of significantly identified proteins	115	104	27
% of secreted proteins	74.8	73.1	88.9
% of fraction-specific proteins using gel electrophoresis	57.1	54.5	43.3
% of fraction-specific proteins using LC	24.1	22.6	35.6

**Figure 7:**
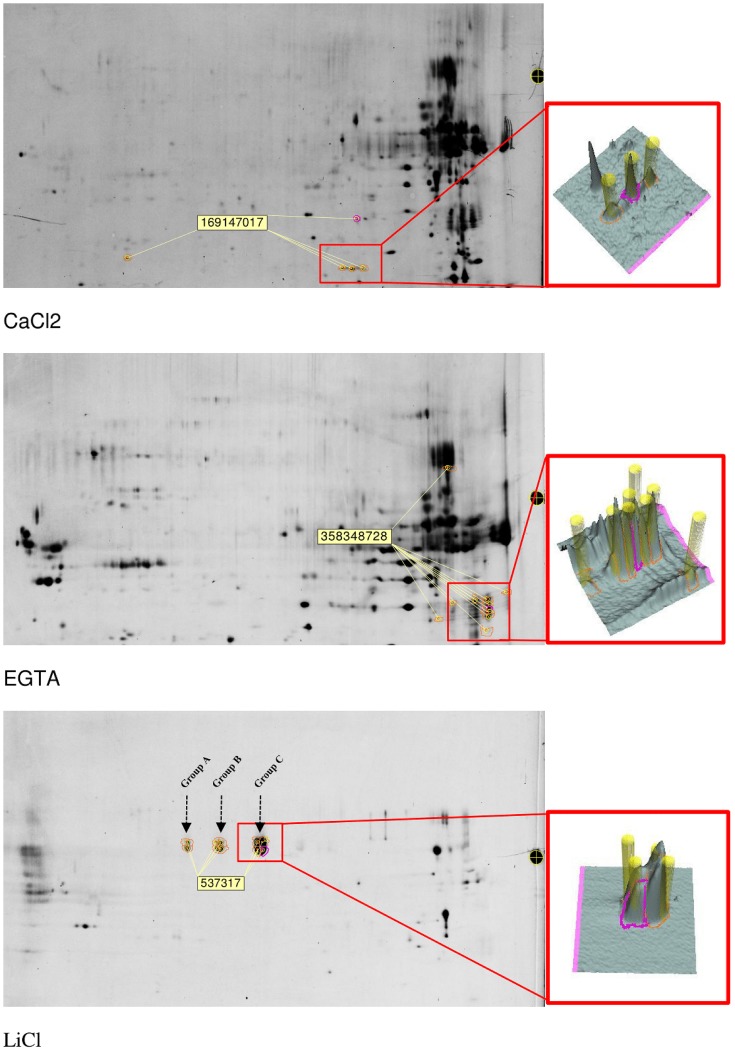
**Localization of some protein isoforms detected after spot detection, picking, digestion, and identification**. In the LiCl fraction, three groups of spots containing peroxidase were identified and arbitrary named group A, B, and C for further MALDI-TOF/TOF characterization.

However, one main benefit of 2D-electrophoresis resides in the possibility to differentiate in one analysis the behavior of various isoforms of the same protein. In each fraction, a panel of proteins was identified in separate spots (Figure 7 and Supplementary Table 3). In the CaCl_2_ fraction, the accession NCBI/gi:169147017, which corresponds to a putative thaumatin-like protein, was found in 5 different spots. The accession NCBI/gi:358348728 attributed to be an alpha-amylase/subtilisin inhibitor was significantly identified in 11 different spots on the gel of the EGTA fraction. In the LiCl extract, the peroxidase NCBI/gi:537317 was identified in 10 different spots than were visibly present in three groups with a different pI.

These groups were arbitrary named “Group A,” “Group B,” and “Group C” as presented in Figure 7. In terms of volumes, these spots in which this peroxidase was identified represent more than 55% of the total volume of all spots visible on the LiCl gels. Since the same protein was automatically assigned in all spots of the 3 groups, the MS and MS/MS spectra were checked, additional peptides were fragmented and manual *de novo* sequencing was performed to determine whether subtle sequence variations could be identified (Table 4). The peptide at 2457 Da corresponds to the peptide **NFDR**QGLDTTDLVALSGAHTIGR and is mainly observed in group C spots. In “Group B” and in “Group A,” the peak at 2516 Da corresponds to the same region of the protein but with the sequence **SNFDK**QGLDTTDLVALSGAHTIGR. Similarly, the peptide at 1025 Da corresponds to the predicted N-terminus of the protein (after removal of the signal peptide), with the sequence QLDNSFY**R**, and is the only form present in the spots belonging to “Group C.” In the “Groups A and B” the same region of the protein is observed as a peak at 997 Da with as sequence QLDNSFY**K**. The same N-terminus, but with a missed cleavage, is confirmed in the peptide at 2276 Da which is not observed in the spots of “Group C.” A peptide at 2591 Da is shared between all groups but a higher signal was recorded for the spots of “group C.” Part of the sequence of this peptide was manually determined as PTL**N**TTYLQTLR with a glycosylation on the Asn-residue. The MS/MS spectrum matches with the determined peptide sequence of a paucimannosidic-type N-glycan HexNac(Fuc)HexNacHex(Xyl)Man(3), a known plant glycosylation structure, on the asparagine (Table 4 and Supplementary Figure 1). None of these differences between the spots explains the observed shift in pI, due to the lack of genome sequence for alfalfa it is furthermore not possible to hypothesize on different functions for peroxidases identified in the different groups.

**Table 4:** **Characterization of three groups of peroxidase identified in the LiCl fraction of the hybrid protocol**.

**Peptide mass (Da)/Peptide sequence**	**Group A**	**Group B**	**Group C**
**997**	**+**	**+**	**X**
QLDNSFY**K** + Gln->pyro-Glu (N-term Q)			
**1025**	**+**	**+**	**+**
QLDNSFY**R** + Gln->pyro-Glu (N-term Q)			
**2457**	**−**	**+**	**+**
**NFDR**QGLDTTDLVALSGAHTIGR			
**2516**	**+**	**+**	**−**
**SNFDK**QGLDTTDLVALSGAHTIGR			
**2591**	**−**	**−**	**+**
PTL**N**TTYLQTLR + HexNac-(Fuc)-HexNac-Hex-(Xyl) Man(3) on **N**			

*Symbols “X,” “−” and “+” refer to the intensity of the peaks of the peptides obtained following MALDI-TOF/TOF analysis. “X”: absence of the peak; “−”: low intensity of the peak; “+”: high intensity of the peak. Modifications in peptide sequence are indicated in bold*.

Manual checking and *de novo* sequencing were similarly performed when high quality peptides spectrum resulted in low scores with the common MASCOT search parameters. In case putative posttranslational modifications were manually identified [HexNAc (N), HexNAc(1)Hex(1) (N), HexNAc(2)Hex(1) (N), …], the MS/MS spectra was submitted again against the NCBInr databases by adding the putative glycosylation events as variable modification in the MASCOT search parameters. If the supposed form of glycosylation however was not listed in the MASCOT posttranslational modifications, the manual identification of the glycosylation was maintained and “putative” was added in the description (Table 5). In our study, the presence of HexNAc residues (with or without additional carbohydrates) on Asn residues was observed in each fraction. Glycosylated CWPs included expansins (or expansin-like), xyloglucan-specific endoglucanase inhibitor proteins, peroxidases, Kunitz-type trypsin inhibitor alpha chain, disease resistance response proteins, low homolog to polygalacturonase inhibitor, receptor-like kinase, and pectinesterase/pectinesterase inhibitor inhibitor 40-like (Table 5). Uncommon glycosylation events were additionally observed on kynurenine, an oxidation product of tryptophan, and on the primary amino group of some peptides (some examples are presented in Table 5 and Supplementary Figure 2). Regarding the switch in molecular mass of 162 Da between the unglycosylated and the glycosylated form of the peptide, an addition of one hexose is likely to have occurred on these residues. There is however no obvious biological significance for this glycosylation in plants, suggesting that these events may result from technical artifacts occurring during the extraction/analysis procedure rather than resulting from an *in vivo* processing of the nascent proteins (Rayon et al., 1998).

**Table 5:** **MALDI-TOF/TOF identification of some glycosylation events detected in the fractions of the hybrid protocol**.

**Spot**	**Fraction**	**Precursor Mass (Da)**	**Sequence**		**Remarks**
334	CaCl_2_		Expansin-like A2-like		
		2375	K.VVLTDLNHNNQTDFVLSSR.A + **HexNAc (N)**		
63	CaCl_2_		Xyloglucan-specific endoglucanase inhibitor protein		
		2915	K.ALNVSTVEPVAPFGTCFASQSISSSR.M + **HexNAc (N)**		
64	CaCl_2_		Xyloglucan-specific endoglucanase inhibitor protein		
		2915	K.ALNVSTVEPVAPFGTCFASQSISSSR.M + **HexNAc (N)**		
220	CaCl_2_		Peroxidase		
		1987	R.IYNETNIDTNFATLR.K + **HexNAc (N)**		
		2134	R.IYNETNIDTNFATLR.K + **HexNAc(1)dHex(1) (N)**		
		2337	R.IYNETNIDTNFATLR.K + **HexNAc(2)dHex(1) (N)**		
415	CaCl_2_		Kunitz-type trypsin inhibitor alpha chain		
		2788	K.GGGLTVANHGENNQTCPLYVVQEK.L + **HexNAc (N)**		
		1545	K.HLALSDQIPSFR.V + **Hex (N-term)**		
371	CaCl_2_		Disease resistance response protein		
		2853	FNGSTLSVLGR + **HexNAc(2)Mannose(8) (N)**	putative	Peptides with 1 and 2 mannose residues less are also present
245	CaCl_2_		Low homology to Polygalacturonase inhibitor		
		2947	LLPNLTGPIPQAIAR + **HexNAc(2)Mannose(6) (N)**	putative	
375	CaCl_2_		Disease resistance response protein		
		2853	FNGSTLSVLGR + **HexNAc(2)Mannose(8) (N)**	putative	Peptides with 7 mannose residues are also present
51	CaCl_2_		Receptor-like protein kinase		
		*2601*	SVVGIQKLNVSYNR + **HexNAc(2)Hex(2)Xyl(1)**	putative	Further addition of HexNAc giving the peaks at 2804 and 3007
267	CaCl_2_		Glucan endo-1,3-beta-glucosidase		
		*2481*	K.VVVSESGWPSDGGFAATYDNTR.V + Trp →**Kynurenin (W) + Hex (Kyn)**	putative	
650	EGTA		Pathogenesis-related protein 1-like		
	–	2461	R.STIISCNYDPPGNYIGQRPF.D + **Hex (N-term)**		
151	EGTA		Probable pectinesterase/pectinesterase inhibitor 40-like		
	–	2737	EITNATEASQFTVR + **HexNAc(Fuc)HexNAcMan(Xyl)Man(2) (N)**	putative	Further addition of HexNAc giving the peaks at 2940 and 3143
280	LiCl		Expansin		
		2710	SLLSNNAAPAGWSFGQTYTGAQFR + Trp →Kynurenin (W) + **Hex (Kyn)**	putative	

*“putative” is added in case the identification has been manually determined and not confirmed by database searching. Glycosylations are indicated in bold*.

The difficulty of resolving basic glycoproteins on 2D-gels has limited the use of this approach to routinely analyze the plant cell wall proteome (Minic et al., 2007; Irshad et al., 2008). It remains nonetheless a powerful approach to elucidate how members of a multigenic family can be differentially translated into proteins. Most interesting is the detection of post-translational modifications such as glycosylations. This is particularly important when studying cell wall proteomes, since N-linked glycosylation is the most prominent modification of secretory proteins (Aebi et al., 2010). In contrast, in LC-approaches proteins are digested prior their separation on column, which often limits the possibility to discern closely related proteins, certainly in a non-model crop such as alfalfa. In our study, more than 85% of all accessions detected to be present in the hybrid protocol were identified by shotgun LC-MS/MS, whereas only 51% were identified using gels, suggesting that shotgun LC-MS/MS should be favored in the mapping of cell wall proteomes, while detailed information on groups of proteins can subsequently be obtained using gel-based approaches.

The analysis of the 3 protocols tested here has highlighted the complementarity of the 3 methods of cell wall protein extraction. The number of proteins that were only identified with the technique developed by Watson et al. (2004) was however relatively low, suggesting that the two other protocols should be preferred when doing global cell wall proteome analyses. In terms of number of identified CWPs, the washes with different sucrose concentrations and the further extraction of the proteins in two- (EGTA-LiCl) or three-steps (CaCl_2_-EGTA-LiCl) gave similar results. The degree of purity of the wall fraction and the yield of cell wall protein extraction varies nonetheless according to the method of extraction. Globally, only the three-steps extraction combines a good purity of the wall fraction and a high yield of protein extraction, two characteristics that favor this protocol for biological studies since the amount of material is often limited. Finally, when the cell wall proteome is divided in 3 sub-proteomes, the complexity of the cell wall extracts is reduced which helps to detect low-abundant proteins.

### Conflict of interest statement

The authors declare that the research was conducted in the absence of any commercial or financial relationships that could be construed as a potential conflict of interest.

## References

[B1] Abdallah C. Sergeant K. Guillier C. Dumas-Gaudot E. Leclercq C. Renaut J. (2012). Optimization of iTRAQ labelling coupled to OFFGEL fractionation as a proteomic workflow to the analysis of microsomal proteins of *Medicago truncatula* roots. Proteome Sci. 10:37. 10.1186/1477-5956-10-37 22672774 PMC3442994

[B2] Aebi M. Bernasconi R. Clerc S. Molinari M. (2010). N-glycan structures: recognition and processing in the ER. Trends Biochem. Sci. 35, 74–82. 10.1016/j.tibs.2009.10.001 19853458

[B3] Albenne C. Canut H. Jamet E. (2013). Plant cell wall proteomics: the leadership of *Arabidopsis thaliana*. Front. Plant Sci. 4:111. 10.3389/fpls.2013.00111 23641247 PMC3640192

[B4] Bozarth C. S. Mullet J. E. Boyer J. S. (1987). Cell wall proteins at low water potentials. Plant Physiol. 85, 261–267. 10.1104/pp.85.1.261 16665667 PMC1054238

[B5] Bradford M. (1976). A rapid and sensitive method for the quantitation of microgram quantities of protein utilizing the principle of protein-dye binding. Anal. Biochem. 7, 248–254. 10.1016/0003-2697(76)90527-3 942051

[B6] Calderan-Rodrigues M. J. Jamet E. Bonassi M. B. Guidetti-Gonzalez S. Begossi A. C. Setem L. V.. (2014). Cell wall proteomics of sugarcane cell suspension cultures. Proteomics 14, 738–749. 10.1002/pmic.201300132 24436144

[B7] Cosgrove D. J. (2005). Growth of the plant cell wall. Nat. Rev. Mol. Cell Biol. 6, 850–861. 10.1038/nrm1746 16261190

[B8] Day A. Fénart S. Neutelings G. Hawkins S. Rolando C. Tokarski C. (2013). Identification of cell wall proteins in the flax (*Linum usitatissimum*) stem. Proteomics 13, 812–825. 10.1002/pmic.201200257 23281244

[B9] Feiz L. Irshad M. Pont-Lezica R. F. Canut H. Jamet E. (2006). Evaluation of cell wall preparations for proteomics: a new procedure for purifying cell walls from *Arabidopsis* hypocotyls. Plant Methods 2:10. 10.1186/1746-4811-2-10 16729891 PMC1524762

[B10] Guerriero G. Hausman J.-F. Cai G. (2014a). No Stress! Relax! Mechanisms governing growth and shape in plant cells. Int. J. Mol. Sci. 15, 5094–5114. 10.3390/ijms15035094 24663059 PMC3975442

[B11] Guerriero G. Sergeant K. Hausman J. F. (2014b). Wood biosynthesis and typologies: a molecular rhapsody. Tree Physiol. 34, 839–855. 10.1093/treephys/tpu031 24876292

[B12] Hühns M. Broer I. (2010). “Biopolymers,” in *Genetic Modification of Plants*, eds Kempken F. Jung C. (Berlin; Heidelberg: Springer), 237–252.

[B13] Irshad M. Canut H. Borderies G. Pont-Lezica R. Jamet E. (2008). A new picture of cell wall protein dynamics in elongating cells of *Arabidopsis thaliana*: confirmed actors and newcomers. BMC Plant Biol. 8:94. 10.1186/1471-2229-8-94 18796151 PMC2551616

[B14] Jamet E. Albenne C. Boudart G. Irshad M. Canut H. Pont-Lezica R. (2008). Recent advances in plant cell wall proteomics. Proteomics 8, 893–908. 10.1002/pmic.200700938 18210371

[B15] Minic Z. Jamet E. Negroni L. rsene der G. P. Zivy M. Jouanin L. (2007). A sub-proteome of *Arabidopsis thaliana* mature stems trapped on Concanavalin A is enriched in cell wall glycoside hydrolases. J. Exp. Bot. 58, 2503–2512. 10.1093/jxb/erm082 17526915 PMC2394711

[B16] Nozahic V. Amziane S. Torrent G. Saïdi K. De Baynast H. (2012). Design of green concrete made of plant-derived aggregates and a pumice–lime binder. Cement Concrete Comp. 34, 231–241 10.1016/j.cemconcomp.2011.09.002

[B17] Pelloux J. Rustérucci C. Mellerowicz E. J. (2007). New insights into pectin methylesterase structure and function. Trends Plant Sci. 12, 267–277. 10.1016/j.tplants.2007.04.001 17499007

[B18] Printz B. Sergeant K. Lutts S. Guignard C. Renaut J. Hausman J. F. (2013). From tolerance to acute metabolic deregulation: contribution of proteomics to dig into the molecular response of alder species under a polymetallic exposure. J. Proteome Res. 12, 5160–5179. 10.1021/pr400590d 24015726

[B19] Rayon C. Lerouge P. Faye L. (1998). The protein N-glycosylation in plants. J. Exp. Bot. 49, 1463–1472.

[B20] Robertson D. Mitchell G. P. Gilroy J. S. Gerrish C. Bolwell G. P. Slabas A. R. (1997). Differential extraction and protein sequencing reveals major differences in patterns of primary cell wall proteins from plants. J. Biol. Chem. 272, 15841–15848. 10.1074/jbc.272.25.15841 9188482

[B21] Sreenath H. K. Koegel R. G. Moldes A. B. Jeffries T. W. Straub R. J. (2001). Ethanol production from alfalfa fiber fractions by saccharification and fermentation. Process Biochem. 36, 1199–1204 10.1016/S0032-9592(01)00162-5

[B22] Verdonk J. C. Hatfield R. D. Sullivan M. L. (2012). Proteomic analysis of cell walls of two developmental stages of alfalfa stems. Front. Plant Sci. 3:279. 10.3389/fpls.2012.00279 23248635 PMC3521126

[B23] Vizcaino J. A. Deutsch E. W. Wang R. Csordas A. Reisinger F. Rios D.. (2014). ProteomeXchange provides globally coordinated proteomics data submission and dissemination. Nat. Biotechnol. 32, 223–226. 10.1038/nbt.2839 24727771 PMC3986813

[B24] Watson B. S. Lei Z. T. Dixon R. A. Sumner L. W. (2004). Proteomics of *Medicago sativa* cell walls. Phytochemistry 65, 1709–1720. 10.1016/j.phytochem.2004.04.026 15276432

[B25] Watson B. S. Sumner L. W. (2007). “Isolation of cell wall proteins from *Medicago sativa* stems,” in *Plant Proteomics*, eds Thiellement H. Zivy M. Damerval C. Méchin V. (Totowa, NJ: Humana Press), 79–92. 10.1385/1-59745-227-0:79 17093305

[B26] Wolf S. Hematy K. Hofte H. (2012). Growth control and cell wall signaling in plants. Annu. Rev. Plant Biol. 63, 381–407. 10.1146/annurev-arplant-042811-105449 22224451

