# Supplementary material for: An improved protocol to study the plant cell wall proteome
Source: Front Plant Sci. 2015 Apr 10;6:237. doi: 10.3389/fpls.2015.00237 (PMC4392696; doi:10.3389/fpls.2015.00237)

Supplementary figure 1. Spectral bands of MS spectra (921 Da to 1059 Da and 2430 Da to 2611 Da) obtained after MALDI-TOF/TOF analysis of each group (A, B and C) of peroxidase found in the LiCl fraction of the hybrid protocol. Peptide sequences were *de novo* determined from MS/MS spectra. Boxes of a same colour correspond to peptides having similar sequences originating from different protein isoforms.

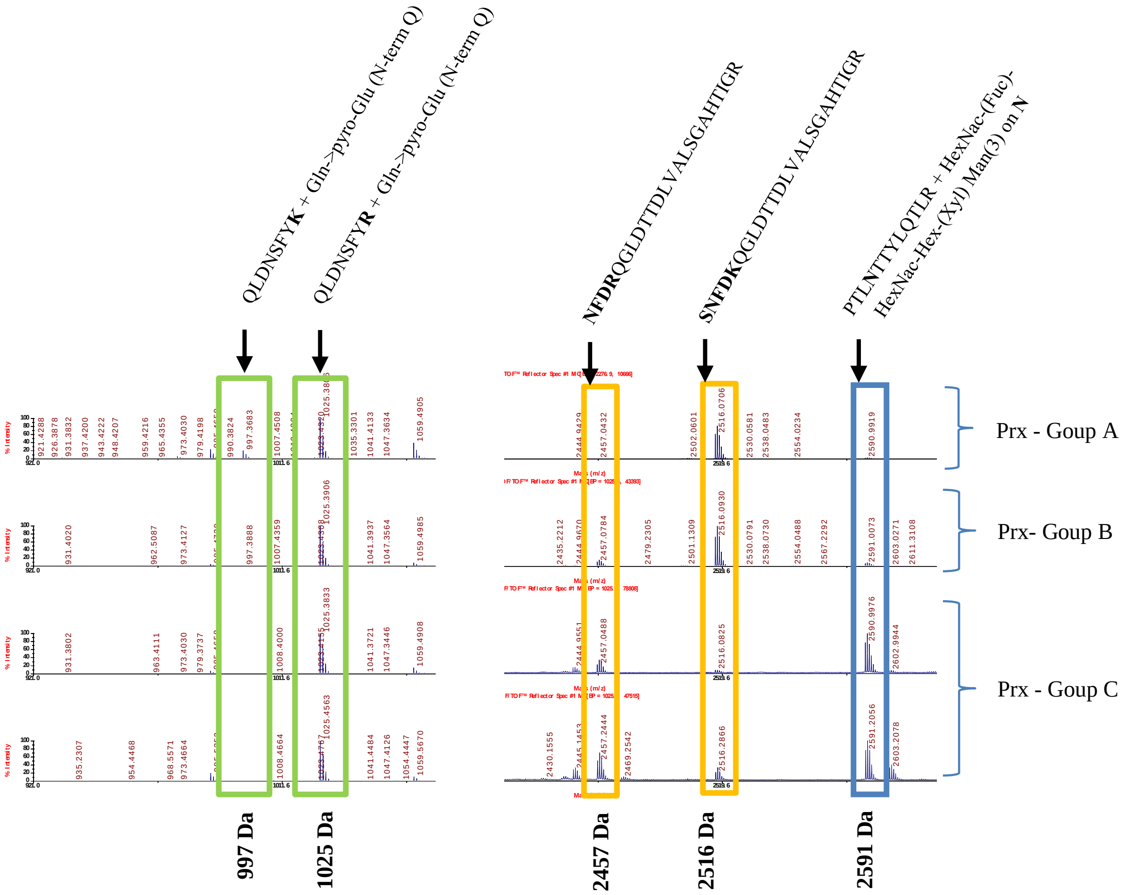

Supplement: Supplementary file 4 [file Image1.PDF]
