# Supplementary material for: An improved protocol to study the plant cell wall proteome
Source: Front Plant Sci. 2015 Apr 10;6:237. doi: 10.3389/fpls.2015.00237 (PMC4392696; doi:10.3389/fpls.2015.00237)
Supplement: Supplementary file 5 [file Image2.PDF]

Supplementary figure 2. Examples of uncommon glycosylation events observed on kynurenine and on the N-terminus end of some peptides

CaCl2 - spot 267 gi|357448997 Glucan endo-1,3-beta-glucosidase [*Medicago truncatula*]

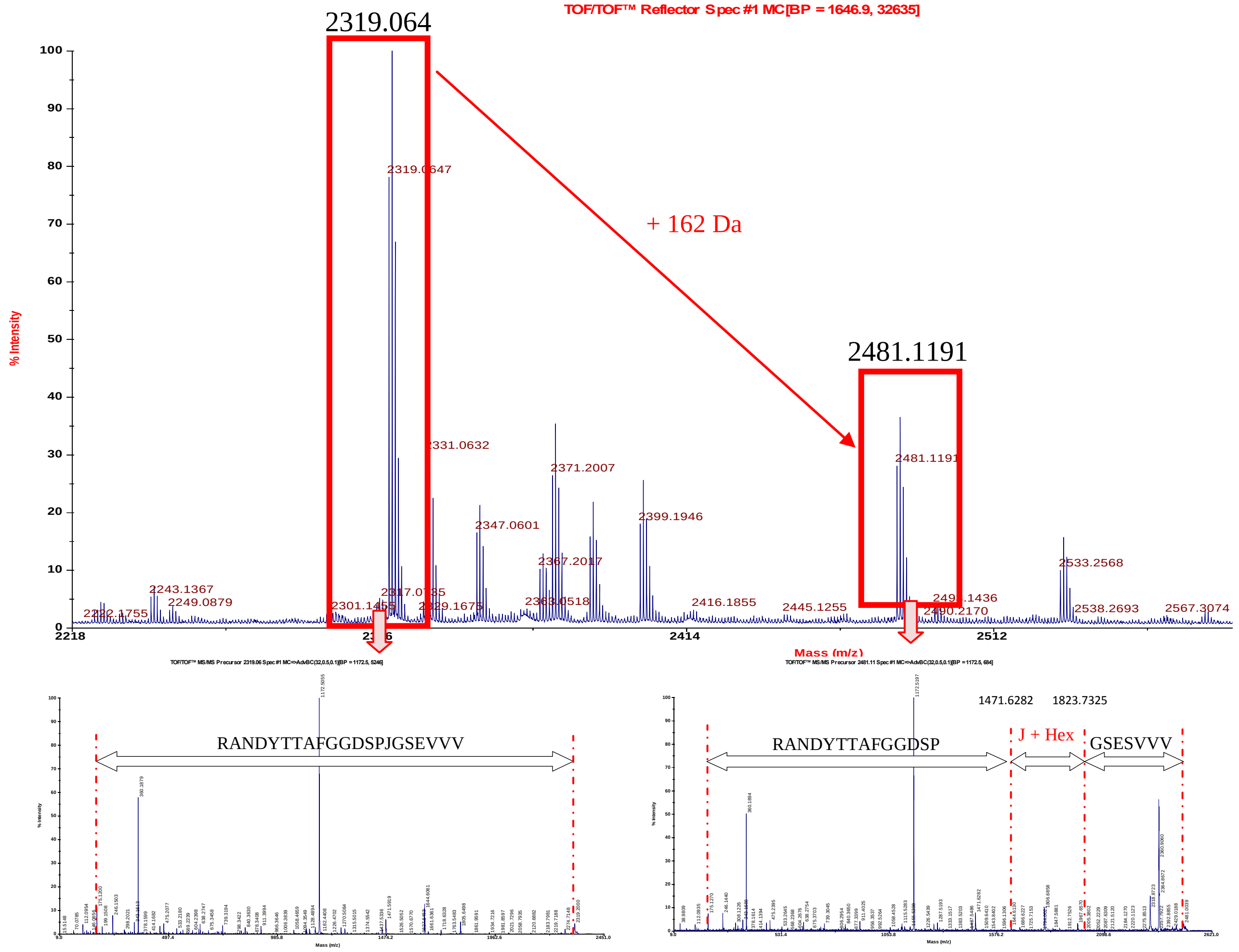

<u>CaCl2 - spot 415, gi|357499761 Kunitz-type trypsin inhibitor alpha chain [*Medicago truncatula*]</u>

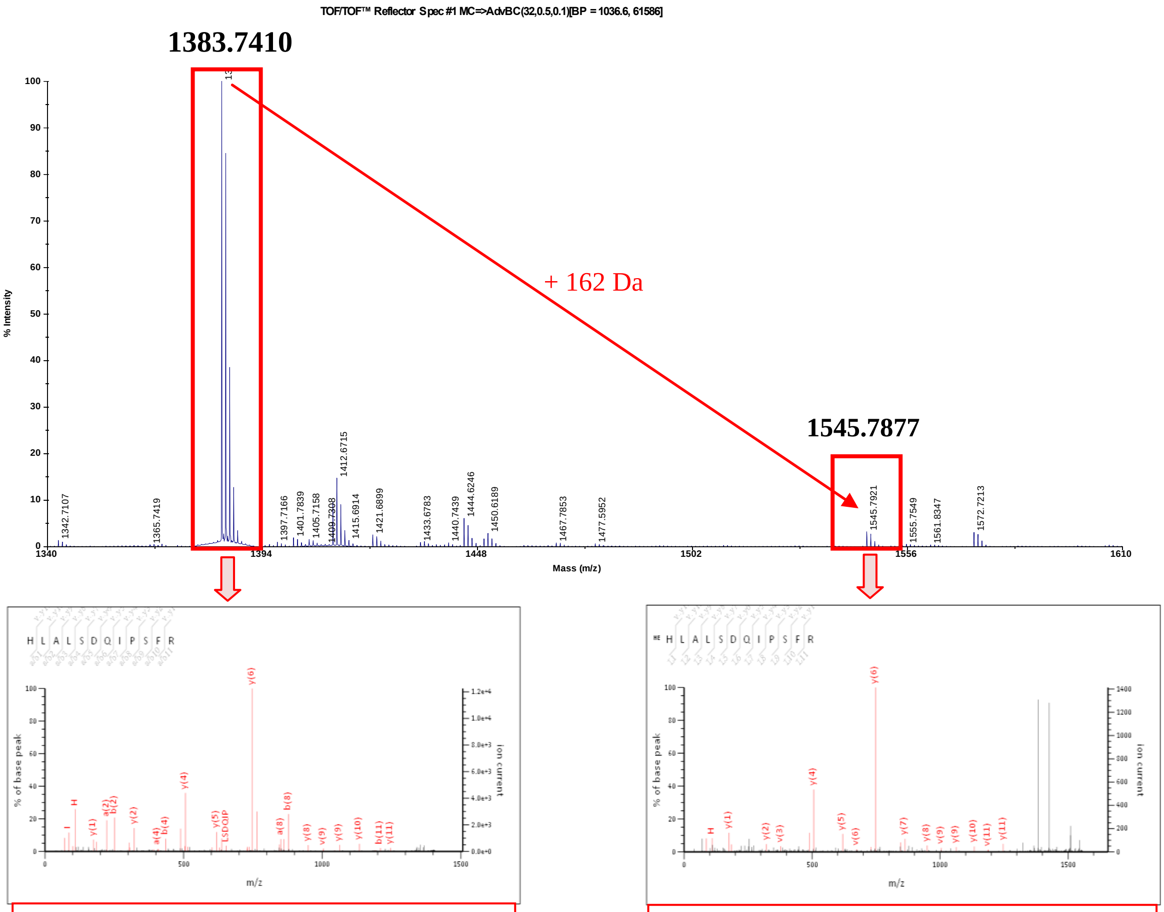

Peptide at 1383.741

K.HLALSDQIPSFR.V

Peptide at 1545.7877

K.HLALSDQIPSFR.V + Hex (N-term)
